# Mesenchymal stem cells in cancer therapy; the art of harnessing a foe to a friend

**DOI:** 10.22038/IJBMS.2021.58227.12934

**Published:** 2021-10

**Authors:** Mehdi Karimi-Shahri, Hossein Javid, Alireza Sharbaf Mashhad, Shaghayegh Yazdani, Seyed Isaac Hashemy

**Affiliations:** 1 Department of Pathology, School of Medicine, Gonabad University of Medical Sciences, Gonabad, Iran; 2 Department of Pathology, School of Medicine, Mashhad University of Medical Sciences, Mashhad, Iran; 3 Department of Medical Laboratory Sciences, Varastegan Institute for Medical Sciences, Mashhad, Iran; 4 Department of Clinical Biochemistry, Faculty of Medicine, Mashhad University of ‎Medical Sciences, Mashhad, Iran; 5 Department of Medical Laboratory Sciences, Mashhad University of Medical Sciences, Mashhad, Iran; 6 Department of Medical Laboratory Sciences, Ilam Institute for Medical Sciences, Ilam, Iran; 7 Surgical Oncology Research Center, Mashhad University of Medical Sciences, Mashhad, Iran

**Keywords:** Carcinogenesis, Mesenchymal stem cells, Molecular targeted – therapies, Neoplasm, Tumor microenvironment

## Abstract

For a long time, mesenchymal stem cells (MSCs) were discussed only as stem cells which could give rise to different types of cells. However, when it became clear that their presence in the tumor microenvironment (TME) was like a green light for tumorigenesis, they emerged from the ashes. This review was arranged to provide a comprehensive and precise description of MSCs’ role in regulating tumorigenesis and to discuss the dark and the bright sides of cancer treatment strategies using MSCs.

To gather the details about MSCs, we made an intensive literature review using keywords, including MSCs, tumor microenvironment, tumorigenesis, and targeted therapy. Through transferring cytokines, growth factors, and microRNAs, MSCs maintain the cancer stem cell population, increase angiogenesis, provide a facility for cancer metastasis, and shut down the anti-tumor activity of the immune system. Although MSCs progress tumorigenesis, there is a consensus that these cells could be used as a vehicle to transfer anti-cancer agents into the tumor milieu. This feature opened a new chapter in MSCs biology, this time from the therapeutic perspective. Although the data are not sufficient, the advent of new genetic engineering methods might make it possible to engage these cells as Trojan horses to eliminate the malignant population. So many years of investigation showed that MSCs are an important group of cells, residing in the TME, studying the function of which not only could add a delicate series of information to the process of tumorigenesis but also could revolutionize cancer treatment strategies.

## Introduction

The human mesenchymal stem is a wide group of stem cells, which attracted attention in cancer research studies due to their ability to change the architecture of the tumor microenvironment (TME). These groups of cells have immunomodulatory properties, they could induce self-renewal in other neighboring stem cells and stimulate tissue regeneration. Besides their multifunctional ability, these cells could also be harvested from different sources such as bone marrow (BM), adipocytes, and peripheral blood, which make them easy-to-read targets for cancer investigations (1). While a considerable number of studies suggested that MSCs might be involved in the process of tumorigenesis, some other investigations showed that these groups of cells could reduce the viability of the tumor cells. The best example of this contradictory mechanism could be seen in the angiogenesis process. In the breast cancer animal models, for example, it has been shown that exosomes derived from MSCs could suppress angiogenesis through miR-16-mediated down-regulation of VEGF (2). However, in the xenograft model, human colorectal cancer cells, it has been claimed that MSCs could deliver IL-6 to the endothelial cells and through activating PI3K/Akt signaling axis promote angiogenesis (3). The same conflict in the results of the studies is also evident in MSC-mediated regulation of cell growth and proliferation. In glioblastoma, ovarian and hepatic cancers, there is evidence suggesting the MSCs-derived microvesicles could suppress the growth and the proliferative capacity of the cancer cells (4, 5). On the other hand, it has been claimed that the primary MSCs migrated to the TME have a hand in increasing the self-renewal capacity of cancer stem cells through producing growth factors such as IL-6 and CXCL8 (6, 7). It seems that based on the component MSCs may carry, they could induce either tumor-suppressive or tumor-promoting ability. It should be noted that the time of MSCs infiltration into the TME might also influence their tumor repressive or progressive activity. It has been claimed that at the primary stages of tumorigenesis, TME might educate the MSCs to alter their gene signature and promote tumor development through either activating tumor metastasis or inducing cell proliferation (8). It has been claimed that the primary MSC infiltration into the TME of ovarian cancer, glioma, and gastric cancer is associated with the elevation in the proliferative capacity and maintenance of stemness in the cancer cells (9-12). This review aimed to provide a comprehensive and precise description of MSCs’ role in regulating tumorigenesis and to discuss the dark and the bright sides of cancer treatment strategies using MSCs.

**Tumor microenvironment**

When it comes to hematologic malignancies, the effect of BM microenvironment on the development, progression, and recurrence of leukemia, lymphoma, and multiple myeloma is undeniable. It has been indicated that the signal transmitted between the neoplastic cells and the cellular components of BM could prolong the survival of malignant cells and could conveniently bypass the cytotoxic effects of many chemotherapeutic drugs. Given this, in many cases, hematologic malignancies’ pathogenesis could not be precisely explained unless an in-depth look has been taken into the BM microenvironment’s role. In solid tumors, conversely, our knowledge about the impact of the TME in disease progression is still minimal (13). 

In a general view, TME, a network comprised of both malignant and non-malignant cells, is a dynamic milieu that is evolved to nourish cancer cells and protect them from devastating cytotoxic signaling whether they are transmitted by immune cells or anticancer agents (14). In plain words, TME is a platform where cancer cells find an opportunity to grow and an armor that shelters cancer cells. It should be noted that TME is composed of a set of cellular and acellular components that have synergistic impacts on each other. Cancer stem cells, innate and adoptive immune-related cells, endothelial cells, mesenchymal stem cells (MSCs), as well as stromal cells are the main cells found in TME (15-17). Each cell has a specific function in the regulation of carcinogenesis. For instance, the residential macrophages in TME, known as tumor-associated macrophages (TAM), widely assist the migration of tumor cells to distant organs (16-18), and more interestingly, they play a protective role against the devastating effects of radiotherapy, anticancer agents, and chemotherapeutic drugs (19-21).

Fibroblasts, another type of cells found in TME, regulate the systematic metastasis of neoplastic cells and go hand in hand with endothelial cells to provide the essential nutrients for tumor cells via angiogenesis regulation. By converting the polarization of the immune microenvironment from a Th2 to Th1 and producing the immunosuppressive cytokines, these cells protect metastatic cancer cells from the immune system (22). Extracellular matrix (ECM), a network consisting of macromolecules, growth factors, and tissue components, is another component of TME, facilitating the communication between tumor cells and other members of TME (23, 24). Apart from these non-malignant cells that gathered to transmit paracrine signals to assist the proliferation, growth, and survival of malignant cells, TME malignant cells could also transmit extracellular paracrine signals or have juxtacrine interactions with immune cells to induce the tolerance phenotype in these residential cells and thereby neutralize the antitumor arm of the immune system (14). Alternatively, tumor cells could also recruit the lymphatic and circulating systems to spread throughout the body and receive the essential nutrients to regulate their metabolic activities. Given these intense interactions between the cancer cells and TME residential cells, a realistic understanding of cancer biology would not be achieved unless we narrowed our view on the biology and function of these non-malignant cells. It seems that many questions and mysteries about the mechanisms involved in cancer recurrence, cancer metastasis, and treatment failure are all going to be answered if our concern is centered on non-malignant components of TME. Table 1 provides brief information about some of the essential functions of non-malignant cells of TME; however, reviewing the precise role of these cells in cancer development is not the topic of the current paper, and herein, we will focus more on the role of MSCS in carcinogenesis and explain how these cells enjoyed unprecedented success in the treatment strategy of cancer. 

**MSCs, essential cells with different faces.**

More than 60 years ago, multipotent stem cells named fibroblast-like cells were harvested from adult BM. Surprisingly, several years after their discovery, it became evident that these cells differentiate into a wide range of mesenchymal lineages such as myocytes, adipocytes, osteocytes, as well as chondrocytes (25-27), and that was when Arnold Caplan *et al.* chose the name of mesenchymal stem cells (MSCs) for the cells (28). Although BM is the original organ that MSCs could be harvested from (1 per 10^5^ of total nucleated cells), these multipotent stem cells could also be found in lower population in other tissues, including the brain, liver, lung, breast, muscle (29-31). According to the site of residency, MSCs might have different intracellular functions. In BM, these cells assist hematopoiesis (32); in adipocytes, MSCs form an immune-evasive environment  (33); and in tissues, MSCs are involved in inflammatory responses and promoting tissue architecture (34-36). Apart from these, a group of MSCs, known as circulating MSCs, also showed a potent tropism to damaged tissues. Circulating MSCs hold a respective share in inducing tissue regeneration and wound healing in cases of injury or infection (36-39). 

As the basic knowledge about the importance of MSCs in tissue homeostasis regulation grew, more studies put these cells under magnifying glass to describe their characteristics more precisely. The *in vitro* studies introduced MSCs as heterogeneous cells with diverse morphological shapes ranging from fibroblast-like, flattened to round cells (40-42). Immunophenotypic studies agree with the morphological studies that declared the MSCs do not demonstrate exclusive cell type-specific markers, which could be due to the cells’ high differentiating ability (43). Although it is complicated to define a specific characteristic for MSCs, these cells are defined as adherent cells that express CD105, CD73, and CD90 (44). 

Apart from all these unique features and broad-spectrum biological functions, do MSCs have a role in tumorigenesis? It seems that the answer to this question not only could revolutionize cancer biology but also could provide a fertile ground for developing novel anticancer treatment strategies. The answer is not as easy as it seems since MSCs appear to have a dual phenotype; immunosuppressive MSCs (MSC-I) and pro-inflammatory (MSC-II). Waterman *et al*. suggested that at the early stages of tumorigenesis, MSC-II could recruit the immune system’s adaptive arm to stimulate tumor surveillance by releasing pro-inflammatory cytokines and chemokines (45, 46). 

The interaction of MSC-II with tumor cells prevents the propagation of some oncogenic signaling pathways such as Wnt and PI3K axes, which may halt cancer cells’ proliferation (47, 48). However, during tumorigenesis, the population of MSC-II is replaced by MSC-I through an unknown mechanism. MSC-I, in turn, provides a mutual interaction with different components of TME and facilitates the condition for cancer cells to grow (15). 

**MSCs, vital stem cells in possession of cancer**

Considering cancer cells as a non-healing chronic wound (49), MSC-I (herein referred to as MSCs) has the potential to be invited to the tumor site by the secreted chemokine/cytokines from TME components. So far, several factors have been identified to be involved in regulating MSCs trafficking and homing. These factors could be categorized into growth factor family (SCF, PDGF, EGF, HGF, and IGF-1) (50, 51), angiogenic family (VEGF, βFGF, and HIF1α) (52, 53), chemokine family (CCL2, CCL5, CCL22, and CXCL12) (51, 54), and last but not least, inflammatory cytokine family (TNF-α, TGF-β, IL-1β, and IL-8) (55-57). Among all, it seems that no interaction could attract MSCs to the tumor milieu more than CXCR4/ SDF-1α axis (58). It should be noted that infiltration of MSCs into the tumor milieu sometimes could be a compensatory mechanism recruited by tumor cells to bypass the cytotoxic effects of treatments. The secretion of TGF-β, VEGF, and PDGF from irradiated breast cancer cells is an excellent example of this evidence. It has been cited that upon the mediators’ production, the expression of CCR2 increased on MSCs, an event that leads to MSCs homing into the tumor milieu (59). 

**Figure 1:**
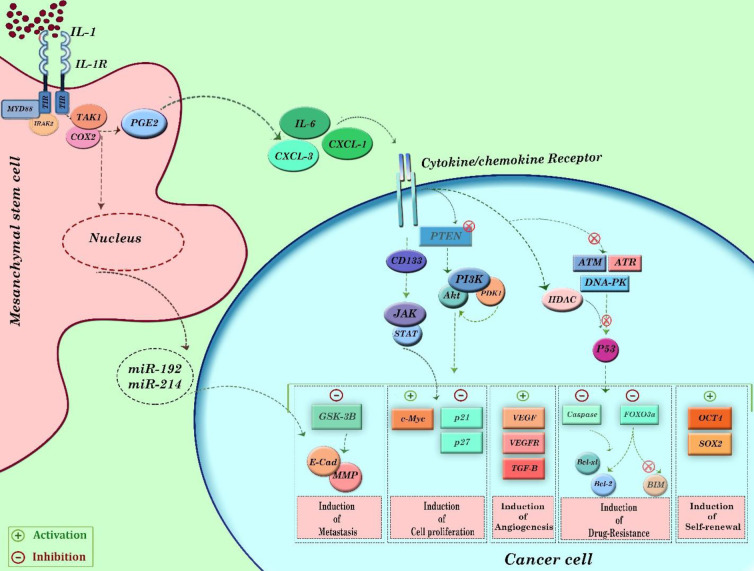
An illustration describing the role of MSCs in cancer development. By producing several chemokines/cytokines or miRNAs, MSCs could propagate different cancer cells' signaling pathways to regulate their survival. As presented, one of the main signaling pathways the stimulation of which is affected by MSCs-cancer cell interaction is the PI3K/Akt axis. As a result of the PI3K/Akt activation, wide alteration in the expression of numerous proteins would occur in cancer cells, which in turn may induce cancer metastasis, angiogenesis, drug-resistance, self-renewal, and cell proliferation. In keeping with the PI3K/Akt signaling axis, other pathways such as JAK/STAT, DNA repair, and histone deacetylase (HDACs) may also be stimulated in cancer cells as a result of MSCs-cancer cells interaction. Activation and inhibition shown by plus and minus signs, respectively

Once arrived, MSCs immediately start to interact with all components of TME. This event subsequently stimulates diverse signaling pathways within MSCs, which leads to production of growth factors, differentiation factors, cytokines, and other tumorigenic factors. For example, in osteosarcoma, MSCs-tumor cells interplay stimulates TGF-β/Smad2/3 axis in MSCs and generates IL-6, which then autocrinally facilitates the differentiation of MSCs into fibroblasts or pericytes. The induced fibroblasts then produced VEGF, a well-known factor that, apart from its participation in angiogenesis, could attract more MSCs into the tumor milieu (60). In gastric cancer, evidence suggested that the stimulated MSCs differentiate into cancer-associated fibroblasts (CAFs) which are notorious for production of SDF-1a in TME, through the TGF-β-dependent mechanism (61). The more produced SDF-1α in TME, the more MSCs would be attracted to the tumor site, reinforcing a vicious circle of tumorigenesis. These findings all shed light on a mutual network between TME components and MSCs that synergistically reinforce the tumorigenesis processes, such as prolonging the survival of cancer cells, inducing metabolic reprogramming, regulating tumor invasion, angiogenesis, counteracting the antitumor immune responses, and sustaining the number of CSCs (62). Some of the most critical roles of MSCs in tumorigenesis’s direct regulation are described in the following part of the paper. Moreover, Figure 1 illustrates some of the roles of MSCs in the development of carcinogenesis.

*Mechanisms through which MSCs orchestrate pro-tumor responses*

The impact of MSCs in tumorigenesis regulation could be reviewed from two perspectives; the effect of MSCs on malignant cells and the influence of MSCs on non-malignant components of TME. 

*Supportive effects of MSCs on cancer stem cells (CSCs)*

Cancer stem cells (CSCs) are responsible for sustaining malignant cells within the tumor site. They are suspected mainly as a driver that orchestrates both tumor metastasis and drug resistance (63). During tumorigenesis and especially in treatment, CSCs gradually differentiate into cancer cells to retrain the bulk of tumor’s cancer cells. Given this important backup function, the tumor niche does its best to maintain the reservoir of CSCs and does not let their numbers drop down (64). MSCs are known as nursery cells that could protect CSCs, enhance their self-renewal and stemness properties by secreting a wide range of mediators, particularly cytokine/chemokines.

It should be noted that the pattern of chemokine production is dependent on the tumor’s type and could be different in each tumor niche (65). IL-6, CXCL1, and CXCL8 are some of the most frequent mediators that are produced from MSCs. Upon interaction of IL-1α and IL-1β with their receptors on MSCs, the expression of prostaglandin E2 (PGE2) will be elevated, leading to IL-6, CXCL1, and CXCL8 secretion (66). The produced IL-6 and CXCL8 eventually up-regulate self-renewal factors, including OCT4 as well as Sox2 in CSCs, and thereby enhance their undifferentiated characteristics (6, 7). IL-6, on the other hand, interacts with CD133 expressed on CSCs and increase their proliferative capacity via stimulating JAK/STAT signaling pathway (67). In some types of cancers, MSCs enhance tumor growth by producing CXCL10, a chemokine that interacts with CXCR3 expressed on CSCs (68). Aside from chemokines, the secretion of bone morphogenetic proteins (BMPs) and several microRNAs such as mir-199 and mir-214 has been attributed to MSCs as a mechanism to sustain CSCs in TME (9, 69).

MSCs could also increase the number of CSCs by altering matrix metalloproteinases’ expression patterns (MMPs). In prostate cancer, for instance, it has been indicated that the CCL5 produced by MSCs increases the expression of MMP9, an event that, on the one hand, could lead to tumor metastasis and, on the other hand, could attenuate the therapeutic efficacy of both chemotherapy and radiotherapy on tumor cells (70, 71). Moreover, sometimes MSCs indirectly increase the survival of CSCs, this time through differentiating into CAFs.  A study reported that MSCs-derived α-smooth muscle actin-positive myofibroblast-like cells prolonged the survival of CSCs in pancreatic cancers through activating the Notch signaling pathway, suggestive of the importance of MSCs in the regulation of cancer metastasis (72). 

*MSCs regulate angiogenesis*

Given the ability of MSCs to release different angiogenesis-stimulating growth factors and cytokines, it is not surprising to bring up MSCs as potent regulators of tumor angiogenesis (73, 74). In pancreatic carcinoma (75), colorectal cancer (3), melanoma (76), glioma (77), as well as many other solid tumors, the footprint of MSCs in the regulation of angiogenesis is evident. Nevertheless, how these groups of cells could increase the micro-vessel density in cancers? One of the best explanations of the mechanism through which MSCs regulate angiogenesis is reported in colorectal cancer. It has been suggested that IL-6 and Angiopoietin-1 (Ang-1) produced by MSCs could stimulate the PI3K signaling pathways in tumor epithelial cells. This signaling axis participates in angiogenesis through up-regulating VEGF and TSP1 (3). Moreover, MSCs increase the structural stability of pre-existing blood vessels (78, 79) through up-regulation of Tie2 (Ang-1 receptor) and Flk1 (VEGFR2) (80). Another mechanism that has been thus far proposed for the angiogenic property of MSCs is mediated through generating other stromal cells. For instance, MSCs-derived CAFs, pericytes, or smooth muscle cells could support endothelial cells’ integrity and enhance tumor blood vessel maturation by releasing both pro-angiogenic cytokines and chemokines, in particular, TNF-α, TGF-β, VEGF, and CXCL12 (81-83). 

*MSCs regulate cancer metastasis*

The participation of MSCs in tumor metastasis can be discussed in three sections; MSCs influence tumor cells motility, MSCs influence epithelial-to-mesenchymal transition (EMT), and MSCs influence the formation of secondary metastatic lesions (44). At each stage, a group of specific mediators aids MSCs in this process. For instance, the secreted C-C and C-X-C type chemokines from MSCs increase cancer cells’ motility (84, 85), and the produced matrix modulating factors such as TGF-β holds a respectable share in regulating EMT(86, 87). Among all mentioned mediators, TGF-β is the main cytokine that transmits the metastatic signal from MSCs to tumor cells. In breast cancer, TGF-β secreted from adipocyte-derived MSCs enhances the motility of cancer cells (87). By binding to its receptors (TGFBR2 ) on tumor cells, TGF-β could up-regulate the expression of *E-cadherin*, *MMPs*, and *rho-associated kinase*, through both Smad-dependent or independent manner (88).

Moreover, the metastatic property of TGF-β is not only restricted to the expression of EMT-related genes, as this cytokine could potentiate cancer metastasis by transferring T helper cells into regulatory T cells (Tregs) via up-regulating FoxP3 (83). Tregs remarkably shield metastatic tumor cells from the immune system’s adaptive arm by inducing immune tolerance (89). TGF-β could also inhibit the anti-tumoral function of natural killer (NK) cells via down-regulating NKG2D and IFN-γ, leading to tumor escape (90).

*MSCs regulates the immune system*

Another function that residential MSCs in TME are notorious for is their ability to secret immunosuppressive mediators. TGF-β, IL-10, TNF-α, and several interleukins are some of the most important mediators through which MSCs paralyze the innate and the adaptive arms of the immune system (91, 92). IL-4 is one of the critical immunosuppressive cytokines that could differentiate naive T cells (T helper 0) (Th0) to Th2, a sub-group of T cells, which could induce anti-inflammatory responses. It should be noted that Th1, in reverse, is the main sub-group of CD4 positive T cells that could stimulate antitumor responses through stimulating both natural killer (NK) cells and cytotoxic T cells. The produced IL-4 from MSCs shifts the equilibrium between Th1 and Th2 toward Th2, an event that leads to suppression of antitumor defense (93, 94). Simultaneously, the production of IL-10 and TGF-β by MSCs prevents the proliferation and stimulation of both NK cells and cytotoxic T cells (102) and increases Tregs’ number in the TME (95, 96). Another mechanism that has been attributed to MSCs in suppressing immune responses in TME is the conferring cytotoxic T cell (CTL) exhaustion, which is mediated through the production of INF-γ. Upon INF-γ secretion, the expression of both inhibitory receptors program death ligand-1 (PDL-1) and CTLA4 increased on cytotoxic lymphocytes.

Moreover, MSCs are the best reservoir of soluble PD-1 ligands, which through interacting with PDL-1 expressed on T cells, attenuate the stimulatory effects of IL-2 by suppressing the PI3K/Akt signaling pathway (44). The expressed CTLA4 on T cells binds to the B7 receptor expressed on antigen representing cells (APCs) and prevents co-stimulatory receptors’ activation (CD28). It should be noted that sometimes MSCs release CTLA4 monomers to hamper the complete activation of CTLs in the tumor milieu (44). Besides T lymphocytes, some evidence suggested that MSCs might have detrimental effects on B lymphocyte proliferation and antibody secretion by inducing cell cycle arrest and overexpressing the *galectin-9* gene (97, 98). The suppressive impact of MSCs in tumor immune surveillance is not always directly. In some cases, MSCs produce CCL2 and increase the trafficking of myeloid-derived suppressor cells (MDSCs), a mechanism that could attenuate the activity of different lymphocytes and NK cells (99). 

**Figure 2:**
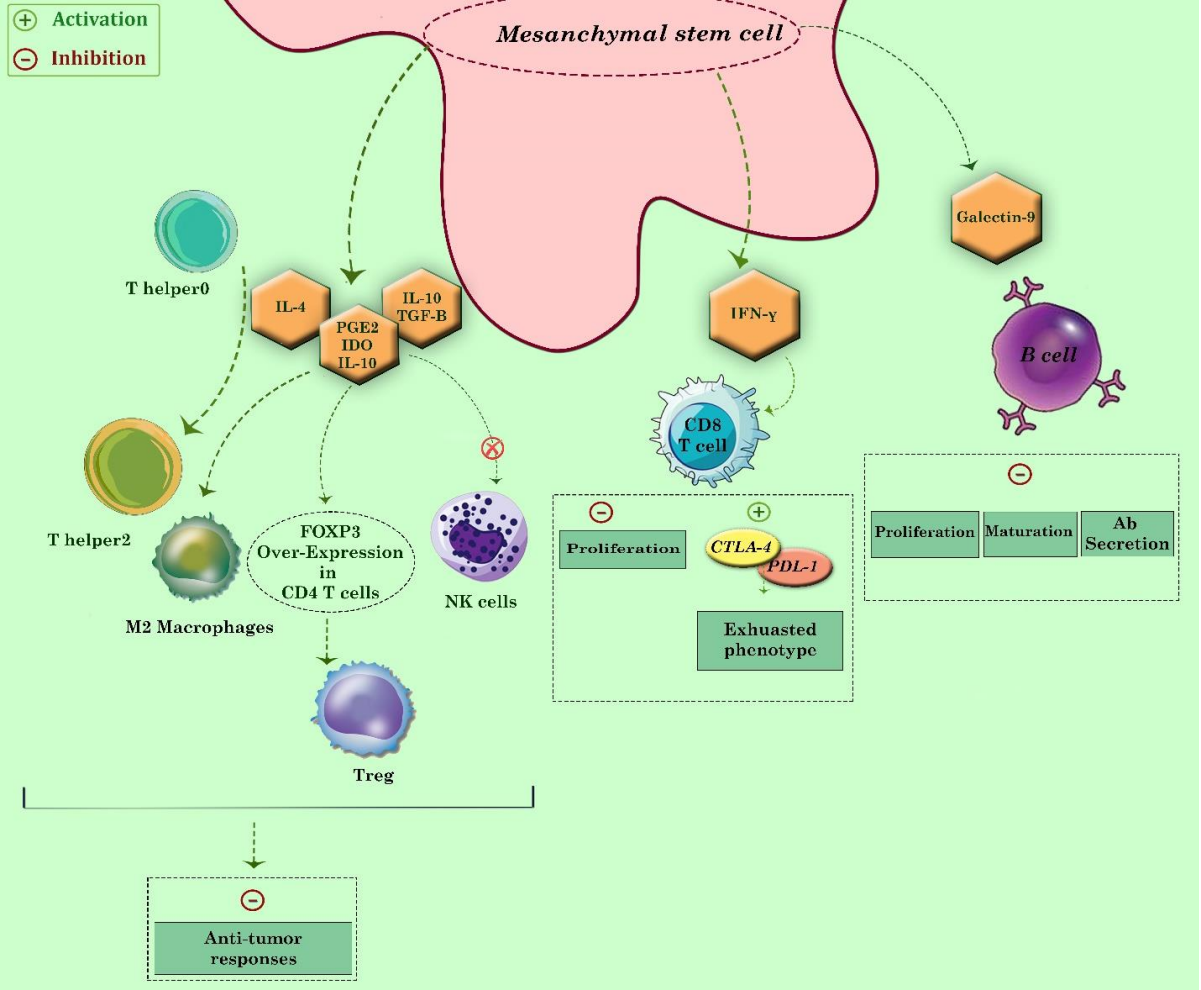
A summary of mechanisms through which MSCs protect cancer cells from the immune responses. As presented, MSCs generate cytokines and mediators, each may have attenuating impacts on the immune cells. Among these mediators, IL-4, IL-10, and TGF-β hold a respectable share in suppressing antitumor immune responses by increasing the population of Th2 and M2 macrophages and through inactivating NK cells. Besides, IL-10 and TGF-β could shut the immune system's adaptive arm down by converting CD4 T cells into Tregs via increasing the expression of FOXP3. In addition to the discussed cytokines, both produced galectin-9 and IFN-γ could also hamper B cells and CD8 positive T cells functions by either preventing their development or induction of exhausted phenotype, respectively. Activation and inhibition shown by plus and minus signs, respectively

When it became evident that MSCs could attenuate the cytotoxic effects of NK cells, much attention was attracted to evaluate whether these groups of stem cells could suppress other members of the innate immunity system. Interestingly, the results suggested the attenuating role of MSCs in the maturation and antigen processing of dendritic cells via stimulating the expression of PGE2 (100). Combining PGE2, IL-10, and Indoleamine 2, 3-dioxygenase (IDO) expression in the TME also converts M1 macrophages to pro-tumorigenic M2 macrophages (101). Taken together, all of this evidence highlighted the immunomodulatory potential of MSCs in TME and suggested why these cells are essential for the survival of neoplastic cells. The impact of MSCs in regulating immune system responses is depicted in Figure 2.

*MSCs regulates the drug-resistant phenotype*

It is well-established that induction of drug-resistant phenotype is the prominent challenge that physicians face in treating different cancer types. Thus far, several mechanisms have been enumerated for the induction of resistance phenotypes, such as perturbation in the mTOR (102), NK1R (103, 104), Wnt (105), PI3K (106), and Notch (107) signaling pathways as well as redox-active proteins (108-110). Given the protective role of MSCs for cancer cells, it is no surprise if these groups of cells confer drug-resistance phenotype to the cancer cells. Through interacting with tumor cells, MSCs could transmit anti-apoptotic signals, activate DNA repair systems, and up-regulate membrane transporters responsible for drug flux such as MDR in malignant cells via activating several oncogenic signaling pathways (111, 112). For example, in breast cancer, adipocyte-derived MSCs could counteract the cytotoxicity of trastuzumab and radiation through suppression of PTEN, the main regulator of the PI3K/Akt signaling axis (113).

Moreover, it seems that the signal transmitted from MSCs could elevate the expression of some epigenetic modulator enzymes such as histone deacetylase (HDACs). This mechanism shifts the balance between pro- and anti-apoptotic proteins in cancer cells in favor of anti-apoptotic protein expression (114). In chronic myeloid leukemia (CML), MSCs seem to suppress the enzymatic activity of caspase-3 and induce resistance in response to imatinib (115). In chronic lymphoid leukemia (CLL), the co-cultured MSCs mitigated the therapeutic potential of Forodesine (116). In solid tumors, the evidence suggested that the paracrine effects of Il-6, IL-8, CXCR4, and IGF might endow cancer cells with the ability to counteract with cytotoxic effects of anticancer agents by up-regulating the expression of *Bcl-2 *and* Bcl-xl* (117-119). Moreover, it has been suggested that in the presence of some chemotherapeutic drugs such as cisplatin, MSCs produce fatty acids that could act as a shield to protect cancer cells (120). Taken together, the number of mechanisms through which MSCs could induce drug-resistance in cancer cells are more than what we discussed here; however, these were some of the most common ways that MSCs used to attenuate the devastating signals of anticancer agents on tumor cells. 

*MSCs from the therapeutic perspective*

The success of MSCs in protecting cancer cells in the TME has made a new scenario in cancer treatment approaches. There is a consensus that probably harnessing these multipotent stem cells could promisingly attenuate the immortalized characteristic of cancer cells and make them more vulnerable to cancer treatment strategies (121). However, the story of applying MSCs in cancer treatment is not restricted to directly targeting these cells, as the recent investigations shed light on another facet of these cells in treating cancer. Initially, the high tropism of MSCs to the tumor milieu could be recruited by the therapeutic approaches to deliver anticancer agents into tumor cells directly. In this case, MSCs could be engineered to be applied as a platform to carry either chemotherapy drugs, oncolytic viruses, or Nano-particles (NPs) into the tumor site (122-124). The second property of MSCs that could be employed in therapeutic approaches is producing nano-sized and lipid-bilayer-enclosed extracellular exosomes. The recent advances in clinical and pharmaceutical approaches make it possible to genetically engineer MSCs-derived exosomes to express the genes. Their products could directly or indirectly induce cell death in cancer cells. For example, engineering MSCs-derived exosomes could allow physicians to increase the number of cytotoxic CD8 positive T lymphocytes in the tumor milieu. This event mobilizes the immune system against the tumor cells (125). The following part of the paper is allocated to discuss the outcome of recent investigations in the field of MSCs-related therapies. It should be noted that some of these therapeutic approaches finally reached the clinical trials. The outcome of some of these trials was summarized in Table 2. 

*MSCs as a tool to carry anticancer drugs*

The high tendency of MSCs to the tumor cells, their low immunogenicity, and last but not least, the safety profile in clinical investigations, all together fueled the interest in using these cells as a promising strategy for anticancer treatments (126). As mentioned earlier, MSCs are a promising platform to deliver chemotherapeutic drugs, oncolytic viruses, and NPs.

*MSCs, a Trojan horse to deliver chemotherapeutic drugs*

The ability of MSCs in delivering anticancer agents has been tested in several investigations in the diverse types of cancers, ranging from solid tumors to hematologic malignancies (127-129). One of the first pieces of evidence suggesting the advantages of MSCs as a Trojan horse for chemotherapeutic drugs was reported in 1999 by Pessina *et al*. They used BM-derived MSCs as a vector for doxorubicin and surprisingly reported that BM-MSCs could sustain the toxicity of doxorubicin and its metabolites against the anti-oxidant components (130). One of the restrictions of doxorubicin in therapeutic approaches is its instability, which leads to the administration of the agent at higher doses, and would be extremely toxic for normal cells. After this achievement, another study also demonstrated that loading doxorubicin on MSCs could inhibit tongue squamous carcinoma cells’ proliferation more vigorously than the drug’s non-loaded form, suggestive of the ameliorating effects of MSCs on the anticancer property of the doxorubicin (131). 

Another chemotherapeutic drug that has been loaded into MSCs was paclitaxel (PTX). This drug is used to treat multiple myeloma (132), ovarian cancer (133), pancreatic carcinoma (134), melanoma (135), and lung cancer (136). Thus far, the success of MSCs-PTX in inducing cell death in cancer cells has been well-demonstrated. For example, in multiple myeloma, researchers delineated that MSCs-PTX remarkably hampers the proliferative capacity of RPMI 8226 cells (137). In keeping with this, the authors also suggested the more vigorous anticancer property of MSCs-PTX in glioma cells and once again shed light on the effectiveness of MSCs as a tool to carry anticancer drugs to the tumor site (138). In a xenograft model of acute lymphoblastic leukemia (ALL), MSC-PTX showed the anti-angiogenic ability mediated by down-regulating some adhesion molecules such as ICAM1 and VCAM1 (128). 

Furthermore, MSCs delivering PTX in the pancreatic model have also demonstrated success in diminishing the size of the cancer cells (139). Cisplatin is another chemotherapeutic drug that shows to have the ability to be loaded on MSCs. A study suggested that cisplatin could be priming on MSCs to induce pronounced antitumor activities (140). 

*MSCs, a stem cell in the hands of oncolytic virotherapy*

Oncolytic virotherapy is a recent breakthrough in cancer treatment strategies that uses oncolytic viruses such as herpes simplex viruses (141), adenovirus (142), and lentiviruses (143) to deliver anticancer agents into malignant cells. Although this strategy seems to be promising, still, several obstacles are on the way to its clinical success. The results of previous studies have declared manipulated MSCs could deliver herpes simplex virus thymidine kinase (HSV-TK) to the tumor site. Researchers treated glioma-bearing rats with MSCs expressing HSV-TK. They suggested that the tumor’s size and progression ability diminished significantly when glioma-bearing rats were treated with VP-MSCs (144). In prostate cancer, MSCs expressing HSV-TK also stimulated cell death in cancer cells without inducing any harmful side effects for normal cells (145, 146). Apart from directly inducing cell death in cancer cells, it has also been indicated that HSV-TK expressing MSCs could also reinforce the therapeutic value of some prodrugs such as fluorouracil (5-FU). The results of the previous investigation reported the ability of HSV-TK expressing MSCs in potentiating the toxicity of the low dose of 5-FC in the prostate cancer xenograft model. This ability suggests that HSV-TK expressing MSCs could directly eliminate the population of neoplastic cells and could be used synergistically with anticancer agents (147). Overall, the opportunity that MSCs are also prone to become oncolytic virotherapy positions has prompted researchers to investigate their potential as the future treatment strategies of human cancers. 

*MSCs, a stem cell in the hands of nano-based cancer therapy*

The potential of MSCs as a delivery system has opened the way of these stem cells into nano-based cancer therapies. The advent of nano-based therapies has made a revolution in conventional treatment approaches to cancer. The high antitumor activity of the NPs, together with their selective behavior against tumor cells, were a ray of hope that finally, a treatment strategy has been found, which may have profound cytotoxicity without any side effects (148-150). However, it did not take long that this flaming hope turned cold due to the low affinity of NPs to the tumor site. Since then, several approaches have been recruited to improve the delivery of NPs to the tumor milieu, and now, it seems that MSCs could be a promising approach.

It should be noted that the absorbance of nano-compounds into MSCs is mediated through several complicated processes, which are well-reviewed in an article (123). From the therapeutic perspective, several studies, so far, have confirmed the delivery efficacy of MSCs in nano-based therapeutic approaches. In a study conducted on lung cancer xenograft models, it has been indicated that loading nano-docetaxel on MSCs could increase the drug’s access to the cancer site, leading to more potent induction of antitumor property (151). Moreover, in metastatic breast cancer cells, it has been indicated that loading quantum dots (QDs) on MSCs enhances the accessibility of QDs to cancer cells and facilitates the uptake of NPs by breast cancer cells (152). In agreement, another study’s results also suggested that MSCs-harboring SiO_2_NP potentiates sensitivity of breast cancer cells to radiation by excessive production of reactive oxygen specious (ROS) (153). Gold nanoparticles (AuNPs) are other nano-based compounds that MSCs have significantly altered their therapeutic value. Loading AuNP on MSCs can result in a 37-fold increased tendency of gold NPs to the tumor site (154). For AlPcS_4_@FNPs also, it has been indicated that MSCs could be a promising system to deliver this NP into the osteosarcoma TME and thereby increase its potent anti-proliferative effects on neoplastic cells (155). Taken together, all these findings highlighted another aspect of MSCs in treating cancer, this time as a tool that can reinforce the therapeutic property of nano-based therapies. 

*MSCs-derived exosomes, a new drug delivery system*

Exosomes, natural cellular components that could carry a wide range of mRNAs, miRNAs, proteins, and lipids, are a new term in cancer treatment approaches. Since exosomes could readily fuse with the target cell and evacuate their cargo into the cells, these lipid-bilayer vesicles seem to be the best weapon to deliver anticancer agents in the TME (156). Given the ability of MSCs to secrete exosomes, it seems that genetic manipulating of these cells could alter the construction of the TME in a way that reduces the survival of neoplastic cells by expressing apoptotic genes (157).

*Exosomes delivering anticancer proteins*

Numerous studies have manipulated MSCs to stimulate apoptosis in neoplastic cells through producing exosomes. It has been suggested that those engineered MSCs with an amplified expression of INF-β could readily deliver this cytokine to cancer cells through lysosomal trafficking. As a result, INF-β could interact with the STAT3 signaling pathway, thereby restricting the cells’ survival and proliferative capacity. Moreover, it has been reported that the exported INF-β could prevent angiogenesis via suppressing the expression of VEGF in TME and meanwhile increase the number of NK cells in the tumor milieu (158, 159). The INF-β harboring exosome could also increase cancer cells’ sensitivity to cisplatin chemotherapeutic agents (160). Enhancing the expression of INF-γ in MSCs is another mechanism that can be recruited to stimulate apoptosis. In glioma cells, the exportation of INF-γ within tumor cells was coupled with cell death induction (161). It has been suggested that MSCs-derived exosome harboring INF-γ could hamper the proliferative capacity in chronic myeloid leukemia cells (162). Another pro-apoptotic gene whose expression could be enhanced in MSCs is tumor necrosis factor (TNF)-related apoptosis-inducing ligand (TRAIL). By inducing caspase-dependent apoptotic cell death, MSCs-derived exosome harboring TRAIL protein could reduce the survival capacity of several cancer cells, including glioblastoma (163), tongue squamous cell carcinoma (164), myeloma (165), lung cancer (166), Ewing sarcoma (167), and mesothelioma (168). Likewise, treatment of hepatocarcinoma cancer cells with MSCs-derived exosome harboring TRAIL could also potentiate cancer cells’ sensitivity to cisplatin (169). 

Apart from cytokines, some studies also tried to increase the expression of some tumor suppressor genes in MSCs. In this vein, researchers enhanced the expression of tensin homolog deleted on chromosome 10 (PTEN) in BM-derived MSCs. They found that these MSCs-derived exosomes could eliminate the tumor cells in glioblastoma-derived cell lines (170). Another study also suggested that the PTEN mRNA-engineering MSCs potently reduced the viability of glioblastoma-derived U251 cells via inducing G1 cell cycle arrest (171). Apoptin is another pro-apoptotic protein that can selectively lyse cancer cells while it has no harmful effects on normal cells. Other researchers treated hepatocarcinoma cell line (HepG2) with engineered MSCs in which the expression of the apoptin gene was amplified. Their results suggested that MSCs-derived exosome, which delivers apoptin protein into HepG2, successfully reduced the cancer cells’ metabolic activity. They also investigated this approach’s impact in HepG2-transplanted nude mice and found that MSCs-harboring apoptin could remarkably diminish the tumor’s size (127). In the xenograft model of lung cancer, it has also been reported that apoptin-modified MSCs repressed tumor progression via inducing caspase-3-dependent apoptotic cell death (172). 

MicroRNAs are another component of MSCs-derived exosomes that seems to have therapeutic potential in cancer treatment strategies. The recent evidence suggested that delivering both miR-124 and miR-145 into glioblastoma cells via MSCs-derived exosomes increased *Sox2* and *OCT4* expression to hamper the proliferation of glioblastoma cells (173, 174). 

*Exosomes are delivering immune-modulatory compounds*

The immunomodulatory property of MSCs is one reason why many studies in the field of cancer immunotherapy have encouraged the recruitment of these cells (175). Whether enhancing or suppressive immune responses, there is no doubt that components that exist in MSCs-derived exosomes could alter the immune system’s activity of different components. This feature could now be employed by immunotherapy approaches to treat most, if not all, human cancers (176, 177). 

ILs are the first and the most important targets that these therapeutic strategies could use to shift the immune tolerance responses of TME into antitumor immune responses. For example, it has been reported that when glioma-bearing rats were treated with genetically modified IL-2 expressing MSCs, the proliferative capacity of the cells was remarkably mitigated, suggesting that the delivered IL-2 into the TME could enhance the cytotoxic immune responses against malignant cells (178). Similarly, IL-12 over-expressed MSCs seem to prevent the growth of ovarian and glioma cancer cells in rats by activating antitumor surveillance (179, 180). Kim *et al*. also narrowed their view on delivering a pro-inflammatory cytokine IL-21 into B-cell lymphoma’s tumor milieu using MSCs. Their results suggested that IL-21/MSCs treated-lymphoma-bearing mice seem to have fewer malignant cells, probably due to increased infiltration of effector T and NK cells (181). In this regard, Hu *et al.* also claimed the immunological effects of IL-21-bearing MSCs in treating the xenograft model of ovarian cancer (179). 

CX3C chemokine fractalkine (CX3CL) is another immune-stimulatory molecule whose anticancer therapeutic effects have been delivered into the tumor milieu via MSCs-derived exosomes. It has been claimed that MSCs-derived exosomes that deliver CX3CL1 could augment the population effector cytotoxic T cells and natural killer cells in TME. This event eventually leads to the repression of tumor growth in xenograft models of lung carcinoma (182, 183). 

*Barriers toward clinical application of MSCs therapy; challenges and future perspective*

Looking down to the information obtained from more than thousands of completed pre-clinical and clinical trials since 2020, it is now more reasonable to discuss the drivers affecting the success or failure of clinical use of MSCs. Despite the efficacy, high tumor tropism, low immunogenicity, and safety, it should be noted that most clinical trials evaluating the potential of MSCs in cancer therapeutic approaches did not reach the higher phases. Recently, the enthusiasm for applying these stem cells has been muted due to clinical outcomes’ heterogeneity. The heterogeneity in the source and biodistribution of MSCs, the diversity in the pharmaceutical activities, and lack of information about the host response are the main factors that have been accused of being involved in the failure of MSCs-based therapies. Overall, MSCs therapy’s challenges could be discussed in three categories: the challenges of manufacturing MSCs, the challenges of MSCs administration, and the challenges of recipient responses (184). 

*Manufacturing challenges of MSCs and future perspective*

The first parameter that could affect the manufacturing of MSCs stems from the fact that according to the tissue origin of MSCs and the donor characteristics, these cells might have different gene and protein profiles (185, 186). Apart from that, the isolation and preparation methods could also affect the ability of MSCs to produce specific types of products (187, 188). These –all together– make the optimizing therapeutic potential of MSCs, which is essential for the validation of clinical trials difficult. To overcome this challenge, some *in vitro* potency assays have been developed to exclude the low potential MSCs and accurately match the MSCs products with the therapeutic perspective that is needed (189-191). Moreover, to collect more homogeneous MSCs, some biomaterial strategies such as expanding MSCs on soft poly (ethylene glycol) hydrogel matrices and 3D culturing systems are developed to guarantee the effectiveness of the used MSCs in therapeutic approaches (192-194). Using pluripotent stem cells (iPSCs) to produce homogeneous MSCs has also gained tremendous attention in this vein. These cells could readily differentiate into MSCs, and their application in clinical trials is safe. This method is very efficient because, with a minimal passage number, many MSCs would be achieved without affecting the therapeutic value of produced MSCs (195, 196). 

CRISPER/Cas9 is another technique that could be recruited to design MSCs for therapeutic approaches (197). Another challenge that influences the therapeutic potential of MSCs is the cryopreservation that can diminish the tropism of these cells to TME. This event could reduce MSCs-cancer cells interaction and decrease MSCs-derived exosomes’ secretion (184). For tackling this problem, some investigations used “rescued culture” for freshly thawed MSCs, a condition in which they culture MSCs for at least 24 hr in recovery cell culture medium before injection (198, 199). 

*Clinical administration challenges of MSCs and future perspective*

Another challenge that could affect the therapeutic value of MSCs is the way that MSCs are administrated to the patients (184). It has been suggested that the pharmaceutical characteristics of MSCs could be altered according to the injection site and the buffer used for injection. For cancer treatment strategies, systematic administration of MSCs intravenous injection (IV) is highly recommended; however, this approach could affect the homing of MSCs to the target site. A reason that could reduce the attraction of MSCs into the tumor milieu is the incidence of inflammatory responses. The incidence of inflammatory responses in patients could be due to the ABO antigens, complement activation, or accumulation of coagulation factors that could hinder transferring MSCs to the TME (184). To overcome this problem, Moll *et al*. suggested administration of anti-coagulant factors such as low-dose heparin (200).

Moreover, it has been suggested that culturing MSCs in non-immunogenic human serum albumin (HSA) culture medium could avoid the incidence of AB antigen discrepancies. Genetic engineering techniques such as CISPR/Cas9 or anti-sense RNAs could also be recruited to either silence the expression of inflammatory factors in MSCs or coating MSCs with heparin to prevent the triggering inflammatory reactions (201-204). The engineered MSCs could also be designed to increase the expression of CD46 (coagulation inhibitor), CXCR4, and MMP (homing CD marker) to enhance their attraction to the TME (184). In addition to these, encapsulating MSCs in either alginate-poly-d-lysine (PDL)-alginate (APA) microgels (205) or magnetically labeling MSCs (206) could also increase the safe delivery of MSCs to TME and increase their residence time in the tumor milieu. 

*Recipient response challenges and future perspective*

The host environment holds a respectable share in determining the therapeutic value of MSCs. The difference in recipient inflammatory responses, immune system activities, and tissue microenvironment structure could suggest why the behavior of MSCs could be distinguished in each individual. So far, several responses have been identified in the recipients that could diminish the therapeutic efficacy of MSCs. For example, in some cases, it has been observed that cytotoxic T lymphocytes might attack IV injected MSCs and induce perforin-mediated apoptotic cell death in these cells (207). Alternatively, these cells could undergo phagocytosis by recipient macrophages within the first 24 hr after injection. Although the phagocytosis of MSCs by monocytes could eventually lead to the induction of immune tolerance against these cells, it should not be forgotten that the reduction in the number of injected MSCs could overshadow their therapeutic potentials (207). The patient’s disease severity also necessitates the administration of high doses of MSCs, which could increase the risk of unfavorable side effects or could lead to mistaken infiltration of MSCs to other organs (184). Given these, it seems that stratifying patients according to their disease severity and the stage could be the most suitable way to reduce the risk of unfavorable responses in the host.

Moreover, the patients’ peripheral mononuclear cells (PBMCs) could be cultured with MSCs before the treatment to investigate whether the patient’s cells could induce apoptotic cell death in MSCs (208). Additionally, companioning some supportive care such as water-soluble vitamin C (209) and vasodilators (210) with IV injection of MSCs could also prevent the incidence of the host’s unfavorable responses. Another mechanism that could prevent the mistaken infiltration of MSCs into other organs could be mediated through increasing the secretion of some chemokines from the target organ. For example, in animal models, evidence showed that irradiation of target organs before exposure to MSCs could make a chemokine gradient through elevating the levels of SDF-1 (211). This event attracted CXCR4-expressed MSCs directly to the desired organ. 

**Table 1:** Function of non-malignant components of TME in tumorigenesis

**TME component**	**Function**	**Reference**
***Cellular components***		
***Non-immune cells***		
Endothelial cells	Protects cancer cells from antitumor immunity. Regulates angiogenesis.	(15)
Fibroblasts	Allows migration of cancer cells to distant organs through producing FSP1. Regulates angiogenesis through VEGFA.	(212-214)
Stromal cells	Regulates tumor cell growth, invasion, and metastasis. Regulates MSCs proliferation.	(23)
MSCs	Regulates interaction between neoplastic cells and TME. Regulates carcinogenesis through producing SDF-1, MCP-1, LL-37, and TGFβ. Releases NO and exosomes.	(215, 216)
BMDC	Regulates tumor growth by producing growth factors and evolving tumor stem cell niche. Regulates angiogenesis by forming tumor vessel formation.	(14)
MDSC	Induces immunosuppression in TME.	(14)
***Immune cells***		
TH17	Promotes tumor growth by producing IL-17, IL-21, and IL-22.	(217, 218)
T regs	Induces tumor progression by regulating immunosuppression in TME. Indicates as poor prognosis marker in patients.	(219)
B lymphocytes (B10)	Regulates tumor survival, metastasis, drug-resistance, and immune escape through TGF-β-dependent conversion of FoxP3^+ ^cells.	(220)
TAM (M2)	Regulates cancer cells' survival, growth, and invasion through producing tissue remodeling molecules, including MMP-2, 9, TNF-α, CXCL10, and IL-1β. Protects cancer cells from cytotoxic effects of radiotherapy and anticancer agents.	(221, 222)
***Non-cellular components***		
CXCL12 and CXCL14	Regulates tumor migration and proliferation through interacting with epithelial cells.	(223-225)
Selectins	Regulates tumor invasion.	(20, 226)
Cadherins	Mediates homophilic bond formation through a calcium signaling pathway.	(227)

FSP1: fibroblast secreted protein-1; VEGFA: vascular endothelial growth factor A; SDF-1: stromal cell-derived factor 1; MSP-1: monocyte chemoattractant protein 1; LL-37: leucine-37; TGFβ: transforming growth factor β; NO: nitric oxide; BMDC: Bone marrow-derived cells; MDSC: Myeloid-derived suppressor cells; TH17: T helper 17; T regs: regulatory T cells; MMP: matrix metalloprotease

**Table 2:** Clinical investigations for evaluating the efficacy of MSCs in the treatment strategy of cancer

**Approach**	**Population**	**Status**	**Phase**	**Outcome**	**Trial number**
MSCs-HSV-TK	Gastrointestinal tumors	Completed	Phase I/II	Evaluates the efficacy of MSCs-HSV-TK.	2012-003741-15
MSCs-IFNβ	Ovarian cancer	Completed	Phase I	Evaluates the safety and the MTD of MSCs-IFNβ in ovarian cancer patients.	NCT02530047
MSCs- MV-NIS	Ovarian cancer	Recruiting	Phase II	Evaluates the side effects of MSCs-MV-NIS.	NCT02068794
MSCs-TRAIL	Lung cancer	Recruiting	Phase I/II	Evaluates the efficacy of MSCs-TRAIL in lung cancer patients.	NCT03298763
MSCs	Liver cancer	Completed	Phase I	Evaluates the effects of MSCs in ameliorating the prognosis of GvHD in liver transplantation of liver cancer patients.	NCT02557724
MSCs	Prostate cancer	Terminated	Phase I	Evaluates the safety of MSCs and cancer homing.	NCT01983709

## Conclusion

MSCs are a group of stem cells that differentiate into diverse cellular lineages and astonishing biological functions. However, in the context of tumorigenesis, the story behind these stem cells is completely different. In TME, MSCs are responsible for orchestrating the survival and proliferative signals into malignant cells. They have an essential role in providing a protective shield for cancer cells by reinforcing the TME structure. Besides the backup roles, MSCs hold a respectable share in regulating cancer cells’ survival, angiogenesis, tumor metastasis, and even induction of drug resistance. Although each of these functions could bring up the idea of targeting MSCs in cancer treatment strategies, two important characteristics that have opened the way of these cells into treatment approaches were their high tropism to the tumor sites and their ability to release exosomes. In fact, these two unique features have made MSCs a good candidate for genetic manipulations and shed another light on the face of these stem cells, this time as a Trojan horse for carrying cytotoxic agents deep to the heart of malignant cells. Although it seems promising, problems and challenges of these therapeutic approaches were encountered that have postponed the clinical application’s approval. However, in the era of bioengineering techniques, there is a hope that someday, MSCs-based therapies would be positioned as the most profitable approach for treating cancer. Until then, more pre-clinical and clinical investigations are required to study the behavior of MSCs in both tumorigenic and anti-tumorigenic perspectives. 

**Study limitation**

In the present review, we made a literature review on both original and review articles published in the last twenty years with the main focus on the role of MSCs in the regulation of tumorigenesis. To avoid any misunderstanding, we did not cover all types of MSCs found in the TME, such as inflammatory MSCs, which could alter the activity of the immune system into the tumor niche. Moreover, we discussed the tumorigenic role of MSCs and their therapeutic options in general and not in the specific type of cancer. Since the behavior of the cells might be different from cell to cell or organ to organ, perhaps it would be better, if the influence of MSCs would be discussed individually in each type of human cancer. It should be noted that MSCs are a new area in cancer research studies and still, little is known about these groups of cells. Most of the studies are still at the pre-clinical levels, so, many aspects of MSCs in tumorigenesis are described according to the results obtained from animal models. Given these, it seems that MSCs have a long way to be established in the process of tumorigenesis and the present study only minimally shed light on the face of these stem cells.

## Authors’ Contribution

The first draft of the manuscript was written by HJ, MKS, ASM, SY and all authors commented on previous versions of the manuscript. Finally, all authors read and approved the final manuscript. SIH was responsible for coordinating the authors, finalized and submitted the paper.

## Conflicts of Interest

The authors declare no conflicts of interest.
